# The effectiveness of aspirin for migraine prophylaxis: a systematic review: Eficácia da aspirina na profilaxia da enxaqueca: uma revisão sistemática

**DOI:** 10.1590/1516-3180.2016.0165050916

**Published:** 2017-01-05

**Authors:** Cristina Pellegrino Baena, Raíssa Campos D’Amico, Helena Slongo, André Russowsky Brunoni, Alessandra Carvalho Goulart, Isabela Benseñor

**Affiliations:** I MD, PhD. Professor, School of Medicine, Pontifícia Universidade Católica do Paraná (PUCPR), Curitiba (PR), Brazil.; II Medical Student, School of Medicine, Pontifícia Universidade Católica do Paraná (PUCPR), Curitiba (PR), Brazil.; III Medical Student, Faculdade Evangélica do Paraná (FEPAR), Curitiba (PR), Brazil.; IV MD. Professor, Hospital Universitário (HU), Universidade de São Paulo (USP), and Coordinator, Service of Interdisciplinary Neuromodulation (SIN), Laboratory of Neurosciences (LIM-27), Department and Institute of Psychiatry, Universidade de São Paulo (USP), São Paulo (SP), Brazil.; V MD. Epidemiologist, Hospital Universitário (HU), Universidade de São Paulo (USP), São Paulo (SP), Brazil.; VI MD, PhD. Professor, Hospital Universitário (HU), Universidade de São Paulo (USP), São Paulo (SP), Brazil.

**Keywords:** Migraine disorders, Headache, Aspirin, Platelet aggregation inhibitors, Therapeutics, Review [publication type]

## Abstract

**CONTEXT AND OBJECTIVE:**

Many researchers have suggested that aspirin prevents migraines. However, the evidence is unclear. The aim of this study was to analyze the available evidence on the effect of aspirin as a migraine prophylactic.

**DESIGN AND SETTING:**

Systematic review, conducted at the Pontifícia Universidade Católica do Paraná, Brazil, and at the University of São Paulo, Brazil.

**METHODS:**

We performed electronic searches in the databases of MEDLINE/PubMed, Embase, WEB OF SCIENCE, the World Health Organization, CENTRAL and OpenGrey, and we also searched manually for interventional studies published before April 2016 that compared the effects of aspirin with a control, in adults. Two authors independently extracted data on the publication, population recruited, intervention (aspirin dosage, follow-up and combined treatment) and main outcomes (frequency, severity and duration of migraine). We evaluated the quality of the studies using the Cochrane risk-of-bias tool.

**RESULTS:**

Our search retrieved 1,098 references, of which 8 met the selection criteria for this systematic review. The total population was 28,326 participants (18-64 years old); most (96%) were men. The dosage varied from 50 to 650 mg/day across the studies. The risk of bias was generally low or unclear. The only outcome for which most of the studies included (6/8) reported a significant reduction was frequency of migraine, which was reduced at an aspirin dosage of at least 325 mg/day.

**CONCLUSION:**

Aspirin can reduce the frequency of migraines. However, the optimal dosage is unclear.

## RESUMO

**CONTEXTO E OBJETIVO:**

Muitos pesquisadores têm sugerido que a aspirina previne enxaquecas. No entanto, a evidência não é clara. O objetivo deste estudo foi analisar as evidências disponíveis para os efeitos da aspirina como um profilático da enxaqueca.

**DESENHO E LOCAL:**

Revisão sistemática, realizada na Pontifícia Universidade Católica do Paraná, Brasil, bem como na Universidade de São Paulo, Brasil.

**MÉTODOS:**

Foram realizadas buscas eletrônicas nas bases de dados MEDLINE/PubMed, Embase, WEB OF SCIENCE, Organização Mundial de Saúde, CENTRAL e OpenGrey. Nós buscamos manualmente estudos de intervenção publicados antes de abril de 2016, comparando efeitos da aspirina com um controle em adultos. Dois autores extraíram independentemente os dados de publicação, população recrutada, intervenção (dose de aspirina, acompanhamento e tratamento combinado) e os resultados principais (frequência, gravidade e duração da enxaqueca). Foi avaliada a qualidade dos estudos com a ferramenta da Cochrane para risco de viés.

**RESULTADOS:**

A nossa busca recuperou 1.098 referências, das quais 8 preencheram os critérios de seleção para esta revisão sistemática. A população total foi de 28,326 participantes (18-64 anos); a maioria (96%) de homens. A dosagem variou entre 50 a 650 mg/dia em todos os estudos. O risco de viés foi geralmente baixo ou pouco claro. O único desfecho para o qual a maioria dos estudos incluídos (6/8) relatou redução significativa foi a frequência de enxaqueca, que foi reduzida com uma dose de aspirina de pelo menos 325 mg/dia.

**CONCLUSÃO:**

A aspirina pode reduzir a frequência das enxaquecas; no entanto, a dosagem ideal não é clara.

## INTRODUCTION

Migraine is a common and debilitating disorder,^1^^,^^2^ ranking as the third most prevalent disorder and the seventh highest specific cause of disability worldwide.^3^ In the Global Burden of Diseases study, migraine was one of eight conditions that affected more than 10% of the population (11.7%) from 2006 to 2013.^4^ In Latin America, a multicenter study conducted in Argentina, Brazil, Colombia, Mexico and Venezuela found that 62% of the participants suffered from headaches, and that the prevalence of migraine among women was 6.1% to 17.4%, while that among men was 2.9% to 7.8%.^5^

Furthermore, several studies have identified a subgroup of patients who experience chronic migraine,^6^^,^ ^7^ in which headache occurs on at least 15 days per month for more than 3 months,^2^^,^ ^8^ with features of migraine headache on at least 8 days per month. Conversely, migraine with a headache burden of less than 15 days per month is defined as episodic migraine.^2^^,^ ^9^ In both forms of migraine, prophylaxis is indicated.^2^^,^ ^10^

Several medications are used to prevent migraine. Specifically, beta-blockers (metoprolol and propranolol)^11^^,^ ^12^ and anticonvulsants (valproic acid and topiramate) are considered to be level A treatments,^10^^,^^12^^,^ ^13^ while antidepressants (amitriptyline)^14^^,^^15^ are regarded as a level B treatment.^11^ Other medications, such as angiotensin-converting-enzyme inhibitors, have not shown the same efficacy. Nonetheless, they have been advocated as second or third-line agents.^16^

Since the 1980s, aspirin has been considered to be a possible migraine prophylactic.^17^ Despite some well-known side effects (e.g. gastrointestinal and renal dysfunction),^18^ aspirin is a possible means for treating migraine, as it is less costly and safer than some other medications, such as beta-blockers and anticonvulsants.^19^^,^ ^20^ However, few studies have explored the effects of aspirin on migraine. Most investigations involving this drug have primarily been designed to evaluate its impact on cardiovascular outcomes.^17^^,^ ^21^ Nonetheless, several such investigations have reported some benefits on migraine. For instance, in the British Doctors’ Trial,^17^ 5,000 healthy male doctors received 500 mg of aspirin daily; migraines were reported significantly less often in the intervention group than in the control group. Similarly, the Physicians’ Health Study^21^ reported that migraine recurrence was 20% lower among men who had received 325 mg of aspirin on alternate days than among those in a placebo group. On the other hand, the Women’s Health Study,^22^ which was also designed primarily to evaluate the cardiovascular outcomes of aspirin use, reported that low doses of aspirin (100 mg) had a small effect on the frequency, severity and duration of migraine among middle-aged women. However, this effect was not significant, perhaps precisely because the effects on migraine were not the focus of the study.^23^

## OBJECTIVE

These conflicting results indicate that the evidence regarding the effects of aspirin on migraines remains inconclusive. For this reason, we conducted a systematic review to analyze the effectiveness of aspirin for migraine prophylaxis.

## METHODS

### Search strategy

We conducted a systematic review of the current literature (published before April 2016) in the following databases: MEDLINE/PubMed, Embase, WEB OF SCIENCE, WHO, CENTRAL and OpenGrey. We searched for studies that used aspirin as a prophylactic to treat migraine. These computer-based searches combined search terms related to the intervention (“aspirin” OR “aspirin/therapeutic use”) and outcomes of interest (“migraine disorders” OR “migraine disorders/prevention and control”) without any language restriction. The search terms were investigated both as controlled vocabulary (MeSH terms), and as free text words in the title and/or abstract. In addition to the electronic searches, we searched the reference list of all studies included and we also searched manually for interventional studies published before April 2016 that compared the effects of aspirin with a control in adults (Table 1).

**Table 1:**
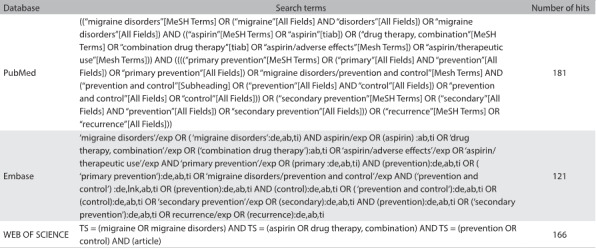
Search strategies

### Study identification and selection

Two authors independently reviewed the title and abstract of each reference to determine whether the study should be included. They based their decision on the following selection criteria. Studies had to:

1. report interventions in the adult population, as randomized controlled trials (RCT) or clinical trials in which an intervention was compared with a control group in a parallel or crossover design;
2. be crossover studies that tested aspirin as a prophylactic treatment for migraine;
3. report the criteria for migraine; or
4. examine the effect of aspirin (acetylsalicylic acid [ASA] or similar) on migraine prophylaxis, regardless of frequency and dose.

Since most studies were published before the Third Classification of the International Headache Society (IHS) defined migraine,^8^ we chose to retrieve all papers that included prophylaxis of migraine as an outcome, regardless of the definition of migraine.

All the studies included reported outcomes within a few hours of the migraine attack. Studies were excluded when:

1. the migraine was described as acute;
2. headache was not differentiated from migraine;
3. the effects of other drugs were compared with those of aspirin;
4. only cost-effectiveness was analyzed;
5. drug therapy was compared with non-pharmacological intervention;
6. pregnant women were included; or
7. animals were used.

Letters, abstracts and conference proceedings were also excluded. Any disagreements regarding article selection were resolved through discussion; a third author was available to resolve disagreements. The papers included were read fully after an initial appraisal. They were then assessed once more by two independent authors to ensure that they met the selection criteria.

### Data extraction

We extracted data using a structured database that had been created prior to the literature search. Specifically, we extracted detailed, study-level characteristics; namely, study design (such as sample size and follow-up duration), population characteristics (age, gender and ethnicity), intervention (aspirin only or aspirin combined with other medications and compared with a control group in which only the other medications were used), outcome assessment (ascertainment criteria), analysis (statistical method, measure of association and sensitivity analyses) and variance (standard error and confidence interval [CI]).

### Quality scoring

Two reviewers independently evaluated the methodological quality of each study. To do so, they used the Cochrane Collaboration tool for assessing the risk of bias in randomized trials,^24^ which categorizes the following domains as “high risk”, “unclear” or “low risk”:

1. random sequence generation;
2. allocation concealment;
3. blinding of participants, personnel and outcome assessors;
4. incomplete outcome data;
5. selective reporting; and
6. other sources of bias.

### Synthesis of results

We had originally intended to perform a meta-analysis that compared migraine frequency between aspirin-treated and placebo-treated patients. However, we were only able to summarize three studies that had reported comparable units of migraine frequency (Benseñor et al.,^19^ Buring et al.^21^ and Bousser et al.^25^). These studies had high heterogeneity (I^2^ = 80.0%; P = 0.007) that could not be explored. Therefore, we chose not to perform a meta-analysis.

## RESULTS

### Study selection

**Figure 1:**
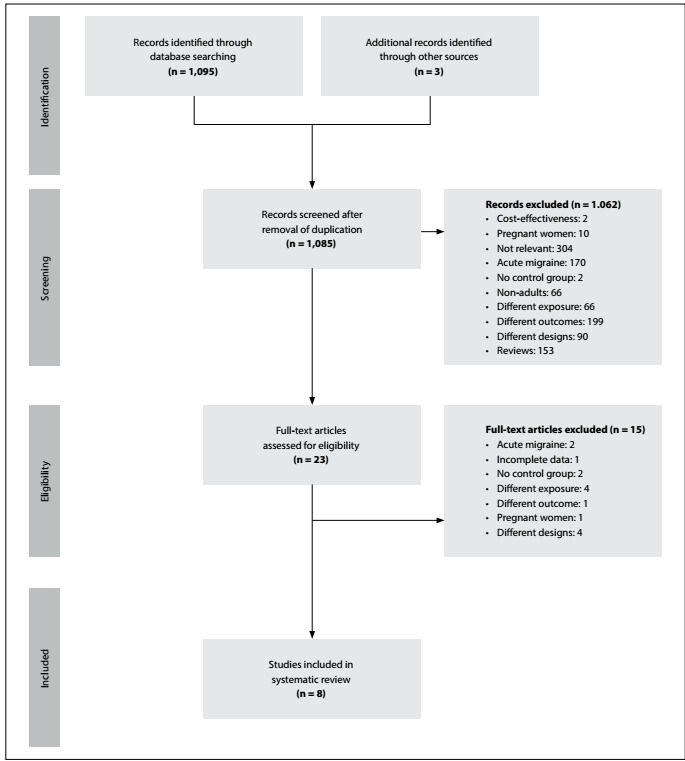
Study flow diagram.

Overall, we identified 1,098 papers, of which 1,062 were excluded on the basis of the title or abstract. The reasons for this exclusion are shown in Figure 1. Most prominently, several studies involved pregnant women or children, some were based on acute migraine and others were designed as reviews, involved a different intervention or evaluated different outcomes. The remaining 23 articles were fully assessed, and eight studies were ultimately selected for data extraction.

### Study characteristics

The characteristics of the eight studies included in this systematic review are shown in Table 2. They included a total of 28,326 participants (sample sizes ranged from 12 to 22,071 participants). Several studies lacked information regarding the number of migraine attacks and the type of migraine. Furthermore, migraine was defined using different criteria across the studies: three studies^25^^,^^26^^,^^27^ defined migraine using the criteria of the *Ad Hoc* Committee on the Classification of Migraine.^28^ Only one study^19^ classified migraine according to the IHS criteria.^29^ Other studies either used their own definition of migraine^30^ or did not mention at all how migraine was defined.^17^^,^^21^^,^^24^ Overall, the studies were designed following two different models: parallel randomized clinical trial^17^^,^^19^^,^^21^ and crossover randomized clinical trial.^25^^,^^26^^,^^27^^,^^30^^,^^31^ Two studies reported an intervention that combined aspirin with other medication (dipyridamole and dihydroergotamine, respectively) and compared this with a placebo.^26^^,^^30^ The remaining studies reported interventions that compared the effects of aspirin with those of a placebo. The ASA dosage used in the studies ranged from low (100 mg every other day)^19^ to high (650 mg every day).^30^^,^^31^ Follow-up periods ranged from 2^25^ to 72^17^ months, with a mean follow-up time of 27.2 months. Five studies included women,^19^^,^^24^^,^^25^^,^^26^^,^^27^ but the two largest studies included in this review only recruited men.^17^^,^ ^21^

**Table 2:**
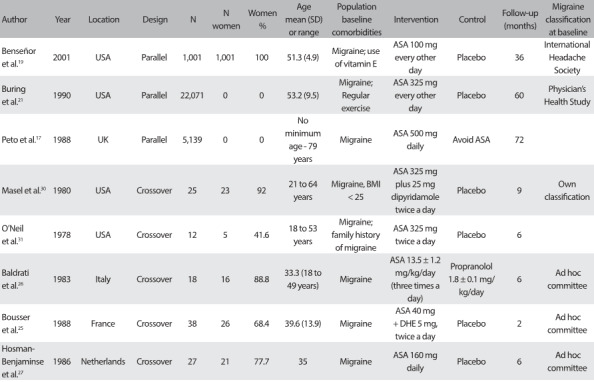
Characteristics of studies included

Table 3 shows the main outcomes reported in the studies included. Frequency, for example, was reported as “migraine attacks per month”,^19^^,^^24^^,^^26^^,^^27^ “migraine index”^26^ and “migraine events per 100,000 men in one year”.^17^ In one study, a migraine index was calculated using the following formula: 1 x (F x D) + 2 x (F x D) + 3 x (F x D), where F is frequency of attacks per month and D is the mean duration of attack in hours.^26^ Severity was reported using different subjective scales;^19^^,^^27^ for instance, 0 = no pain, 100 = severe pain. Two studies that used such scales of measurement also reported the duration of migraine attacks.^19^^,^^25^

**Table 3:**
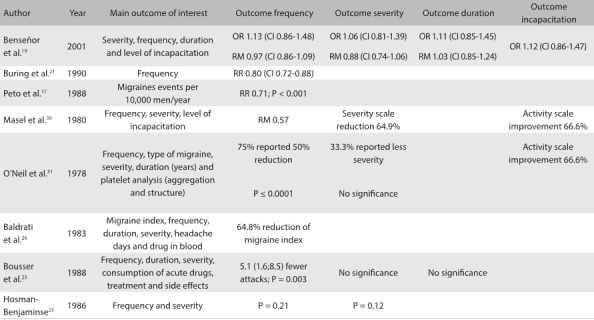
Main outcomes reported in the studies included

OR = odds ratio; CI = 95% confidence interval; RM = risk of migraine; RR = relative risk.

### Characteristics of study populations

Our systematic review included an adult population totaling 28,331 participants. The mean age across the studies ranged from 18 to 64 years, and 96% of the total population (27,218 participants) were men, mainly because two major studies included in the systematic review (the Physician’s Health Study and the British Male Doctors’ Study) consisted of solely male populations. On the other hand, five of the studies recruited mostly women for the interventions.^19^^,^^25^^,^^27^^,^^28^ All the studies reported on otherwise healthy participants.

### Quality assessment of the studies included

**Figure 2:**
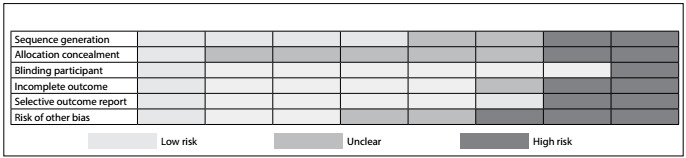
Risk of bias in the studies included.

With regard to random sequence generation, half of the studies showed a low risk of bias. Concerning allocation concealment, only one study had a low risk of bias; most of the remaining studies were determined to have an unclear risk of bias in this regard. In terms of blinding of participants, personnel and outcome assessors, seven studies showed a low risk of bias, and only one had a high risk of bias. Regarding incomplete and selective outcome reporting, most studies showed a low risk of bias. Finally, with regard to the other risks of bias, three studies had a low risk of bias, two showed an unclear risk and three revealed a high risk of bias. The risk of bias in the studies included is presented in Figure 2.

### The effectiveness of aspirin for prophylaxis of migraine

Benseñor et al.,^19^ Buring et al.,^21^ Peto et al.,^17^ O’Neil et al.,^31^ Baldrati et al.^26^ and Hosman-Benjaminse et al.^27^ reported on aspirin as a single active treatment for migraine. All these studies, except for that of Benseñor et al.,^19^ reported that there was an inverse association between aspirin use and migraine frequency.^17^^,^ ^21^^,^ ^24^^,^ ^25^ In studies that found a reduction in migraine frequency, the dosage ranged from 1,300 mg^21^ to 4,550 mg weekly.^31^

Benseñor et al.,^19^ Baldrati et al.^26^ and O’Neil et al.^31^ analyzed the severity of migraine attacks. Only Baldrati et al.^26^ reported that there was an inverse association between severity and aspirin use. Benseñor et al.^19^ and Baldrati et al.^26^ reported on the duration of migraine episodes as an outcome. Baldrati et al.^26^ found an inverse association, while Benseñor et al.^19^ found a direct association that was not significant. Benseñor et al.^19^ was the only study that described incapacitation as an outcome; after having restricted the analysis to women who fulfilled the modified IHS criteria for migraine, they reported that there was a significant improvement in incapacitation after 12 months (OR = 1.45; 95% CI = 1.04 - 2.02).

Masel et al.^30^ reported on an intervention combining dipyridamole and aspirin, while Bousser et al.^25^ combined dihydroergotamine with aspirin as an active prophylactic treatment. Each study reported different doses of aspirin, and both compared the outcomes with those of a placebo group. Both studies reported a decrease in the frequency of migraine episodes. However, neither study showed that aspirin had any significant effect on the other outcomes, such as severity and duration, and neither of them showed any worsening of any of the outcomes reported. Importantly, because the three studies that reported comparable units regarding frequency of migraine (Benseñor et al.,^19^ Buring et al.^21^ and Bousser et al.^25^) had high heterogeneity (I^2^ = 80.0%; P = 0.007), we chose not to perform a formal meta-analysis. The other studies included presented frequency outcomes as a proportion of the study groups’ reported reduction in migraine attacks.

## DISCUSSION

The present systematic review included a total of eight articles reporting the effects of aspirin on different migraine-related outcomes, including severity, frequency and duration. In total, we found consistent reports showing that continuous use of aspirin affects the frequency of migraine episodes. Additionally, we found that higher dosages were associated with better results.

The total weekly dose of aspirin (1,300 mg to 4,550 mg) was higher in studies^17^^,^^21^^,^^24^^,^^25^ that reported that there was an inverse association between migraine frequency and continuous use of the drug than in studies that reported that there was no significant effect.^19^

Frequency was the only outcome that was analyzed in all the studies included. Nevertheless, it was defined and interpreted differently among the studies, which hindered synthesis of our data.

Severity and duration were defined and registered differently; thus, it was difficult to summarize the data. Disability level, necessity for relief drugs and days with headache were isolated outcomes that were only reported in some studies. Therefore, we could not properly assess these data and include them in this systematic review. Finally, because the outcome measurements were so heterogeneous, we were unable to perform a meta-analysis.

There was no significant association between aspirin and migraine. Neither aspirin dosage nor combination with other medications decreased the severity or duration of migraine attacks in the studies included. Nonetheless, few studies reported severity and duration as outcomes, so it is likely that the data were insufficient to address these questions.

The only study to report an inverse association between aspirin and all three main outcomes^26^ also showed high risk of bias. However, the three highest-quality studies showed a significant association^17^^,^^21^^,^^24^ between continuous use of aspirin and reduction in the frequency of migraine attacks, with no significant effect on the duration and severity of outcomes. It is important to note that two of these studies^17^^,^^21^ were designed to ascertain cardiovascular outcomes and that they used higher dosages of aspirin for this reason. This may explain the significant effect on migraine.

Despite earlier interest in aspirin as a possible prophylactic for migraine,^20^ studies comparing aspirin with a placebo in this regard are rare. One strength of the present systematic review is that it gathered individual studies that have tested the prophylactic effect of continuous aspirin use on migraine. Even though most studies had a primary outcome of interest other than migraine, we were able on the basis of the available evidence to identify the direction of association, as well as to ascertain a cutoff dosage for the effect of aspirin on migraine frequency. Furthermore, given that most studies focused on cardiovascular outcomes, we expect that populations using aspirin to prevent cardiovascular events have a lower frequency of migraine.

Our study had some limitations that should be considered. Most importantly, we were unable to classify the migraines that were reported in the studies included according to the recent IHS^8^ definition: we only found primary studies that used very different criteria to define migraine. Additionally, the reporting of outcomes and dosage was not standardized across studies, thus preventing us from performing a formal meta-analysis. Furthermore, because migraines were not classified in the studies included, we were unable to categorize the migraines. Therefore, our results should be applied to the general population with caution. Finally, the use of diverse criteria to define migraine across studies may have introduced some misclassifications or misdiagnoses of migraine. However, we cannot be certain of this, and the prophylactic effect of aspirin on migraine may consequently have been underestimated.

Although we could not gather information regarding quantitative effects, it was possible to identify the direction of association in relation to migraine frequency. With regard to severity and duration, no evidence supports prescription of aspirin for this purpose.

Since other combinations of treatments involving aspirin have recently been tested^32^ as prophylactic treatment for migraine, we believe that the effect of aspirin in isolation needs to be quantified and made known. For effective prophylaxis, the dosage should be more than 325 mg/day: smaller doses did not show significant effects across all studies included. With regard to side effects in this area, dyspepsia, peptic ulcer, upper gastrointestinal bleeding and renal dysfunction should be assessed.

## CONCLUSION

In conclusion, the present systematic review presented the available evidence on the prophylactic effect of aspirin in relation to migraine. The effects on attack frequency were consistent across most of the populations studied, even though the investigations focused on cardiovascular outcomes. Aspirin can reduce the frequency of migraines. However, the optimal dosage is unclear.
